# Supplementary material for: An immunoPET probe to SARS-CoV-2 reveals early infection of the male genital tract in rhesus macaques
Source: Res Sq. 2022 Apr 8:rs.3.rs-1479315. Preprint. [Version 1] doi: 10.21203/rs.3.rs-1479315/v1 (PMC8996619; doi:10.21203/rs.3.rs-1479315/v1)
Supplement: Supplement 23 [file aecf5ffb16b7a822c649e1cc.docx]

CoV2_2 Supplementary Figs and movies List

**LP14:**

S1-Video: LP14 spinning monkey.

S2-video: LP14 organ scan rotation.

S3-video: LP14 lung isolation series.

S4-video: LP14 abdomen PET/CT rotation

S5-video: LP14 abdomen green PET (no CT) rotation

S6-video: LP14 Isolated MGT rotation

**IN22:**

S7-video: IN22 3hr rotation

S8-video: IN22 21hr rotation

S9-video: IN22 organ-1 rotation

S10-video: IN22 organ-2 rotation

S11-video: IN22 lung isolation rotation

**JF82:**

S12-video: JF82 WB wk1

S13-video: JF82 WB wk2

S14-video JF82 overlay

S15-video JF82 organ-1 rotation

S16-video JF82 organ-2 rotation

S17-video JF82 lung isolation rotation

S18-video JF82 MGT isolation rotation

S19- Lung montage.

S20-video MGT montage

S21-video Liver/Heart series

S22-Video prostate series
